# Relationship of autistic traits and the severity of fear of the COVID-19 pandemic in the general population

**DOI:** 10.3389/fpsyt.2024.1260444

**Published:** 2024-02-26

**Authors:** Dominika Bieczek, Adrianna Ściślicka, Agnieszka Bobowska, Filip Tomsia, Krzysztof Maria Wilczyński, Małgorzata Janas-Kozik

**Affiliations:** ^1^ Students’ Scientific Society, Department of Psychiatry and Psychotherapy of Developmental Age, Medical University of Silesia, Katowice, Poland; ^2^ Department of Psychiatry and Psychotherapy of Developmental Age, Medical University of Silesia, Katowice, Poland; ^3^ Department of Psychiatry and Psychotherapy of Developmental Age, John Paul’s II Pediatric Center, Sosnowiec, Poland

**Keywords:** autism spectrum disorder (ASD), fear, COVID-19, autistic traits, attention switching

## Abstract

**Background:**

The aim of the study was to investigate the level of fear of the COVID-19 pandemic and to detect a possible correlation between the autistic traits and the level of fear and to learn about other factors that may affect the level of fear.

**Methods:**

The study utilised a questionnaire and was conducted online in the period from 16.02.2021 to 11.06.2021. The test group consisted of 214 respondents with an average age of 23.78 years (95%CI: 22.48 – 25.08; max: 61, min: 14) from the general population. The study used The Autism-Spectrum Quotient (AQ) questionnaire to assess the degree of autistic traits in the general population and The Fear of COVID-19 Scale, which was used to assess the level of fear of COVID-19.

**Results:**

Among the respondents, 9 people scored ≥32 on the AQ test and were considered to have a high degree of autistic traits. In multiple regression (R^2^ = 0.1, p<0.0001), a positive relationship between the severity of fear of COVID-19 and the autistic traits (p=0.01) and age (p<0.001) was obtained. Additionally, a second multiple regression (R^2^ = 0.1, p<0.000001) including the subscales of AQ was performed and a positive relationship between the severity of fear of COVID-19 and the difficulties in attention switching (p=0.0004) and age (p=0.00001) was obtained.

**Conclusion:**

People with higher autistic traits present greater fear of the COVID-19 pandemic. We suggest that it might be caused by cognitive stiffness and disorders in emotions regulation, according to the literature. The elderly also present higher levels of fear. The other variables did not affect the level of fear of the COVID-19 pandemic.

## Background

The emergence of COVID-19 infections in 2019 in the city of Wuhan began a cycle of huge changes in our daily lives, their effects are visible in all areas – from private to professional life. The dynamic changes of the situation contributed to the announcement of the COVID-19 pandemic by the World Health Organization (WHO) on 11 March 2020 (1). The hitherto unknown virus quickly began to spread around the world, leading to the establishment of epidemic states in most countries of the world. The global health system faced the enormous challenge of preventing the spread of the pathogen and introducing an effective defence strategy (2).

Unfortunately, the pace of spreading the pandemic and the wide range of clinical symptoms ranging from asymptomatic to severe acute respiratory syndromes and multi-organ disorders have had a negative impact not only on physical health (3). The implemented methods to prevent the spread of SARS-CoV-2, including, among others, mass school and workplace closing, have disrupted everyday functioning and changed the concept of a normal lifestyle. The sudden need for social isolation, worrying about one’s own health and safety, and growing unemployment as a result of the introduced restrictions have led to the mental imbalance of many people (4). An increase in the occurrence of dysfunctional behaviours, emotional stress, defensive reactions such as aggression, fear, anxiety, frustration, depression and suicidal tendencies was observed (5). It has been shown that the severity of emerging mental health disorders may vary depending on the studied population. Older people (6), children (7) or health care workers (8) may report varying levels of severity of the disorders. Particularly noteworthy are psychiatric patients, whose level of anxiety may be multiplied as a result of incorrect interpretation, for example, of media coverage. People with autism spectrum disorder; (ASD) present problems in the field of selected cognitive functions, among others, speed of information processing, verbal memory, reasoning or problem solving, as well as interpreting the information that they reach (9). Sensationally charged information about COVID-19 in the mass media could significantly hinder adjusting the subjective sense of threat to its actual degree by people with ASD.

Since the beginning of the COVID-19 pandemic, there have been many articles published that have described the experiences of people with various neurodevelopmental disorders with the regulation of emotions in response to stressful stimuli. The authors drew attention to the problems arising from the loss of routine due to the introduction of new regulations aimed at avoiding the spread of infection (10). In addition, patients with neurodevelopmental disorders have a higher risk of developing mental illnesses. Already at the very beginning of the pandemic, parents of children with neurodevelopmental disorders reported increased anxiety and increasing mental problems of their children (11). Studies conducted so far have shown a higher level of anxiety related to the COVID-19 pandemic in people with neurodevelopmental disorders compared to the general population (10, 12). Loss of routine was a strong predictor of increased anxiety in people with ASD (12, 13).

The basic features of ASD are persistent difficulties in social communication, establishing and maintaining relationships and the occurrence of repetitive and stereotyped patterns of interests and activities (14). The functioning of autistic people in terms of social development can be very diverse. Impeded social interactions are manifested by difficulties in non-verbal communication and development of peer relationships. They are also characterised by difficulties engaging in emotional reciprocity and difficulties with sharing a common field of attention by sharing joys, interests, or achievements with other people (15). Autistic people feel threatened when their environment changes, as they have varying degrees of adaptability (16). Autistic people tend to misregulate their emotions by using maladaptive strategies, such as isolation, information avoidance or excessively searching for information, rumination, suppression of expression of emotions, aggressive or repetitive behaviours. Better strategies are sharing one’s thoughts about stressful situations and distraction that is connected with shifting one’s attention to something else in order to avoid unwanted emotions. The best strategies are cognitive reappraisal, focusing on the positive and humour (12).

Another important issue is the prevalence in the population of a constellation of subclinical features of autism spectrum disorder known as the Broad Autism Phenotype; (BAP). Autism is a continuum of various symptoms that are present in the general population. Everyone has different levels of these features. In between the dichotomous division between the population that has received a diagnosis and the population that does not have enough traits to get one, there are people who present only few traits. As an example of such people is the phenomenon of BAP that was initially analysed in the context of siblings of people with ASD (17, 18), but currently there are general population analyses available in the literature, which determine the frequency of BAP at 9 (19) to even 25% (20) of the population. People from the BAP group present the features of ASD primarily related to the area of social cognition disorders, among others, in the interpretation of facial expressions or theory of mind (21, 22).

Difficulties in adapting to changes raise the question of whether the autistic traits had an impact on the level of fear of the COVID-19 pandemic. For example, a study of the reactions of people injured after the earthquake in L’Aquila in Italy showed that people with higher autistic traits tended to have an inadequate assessment of the situation and denied the need for emotional support (23). In addition, ASD is often accompanied by alexithymia, which intensifies difficulties with the interpretation of internal feelings and with their proper reception and expression (24). Autistic people tend to use patterns to avoid emotional stimulation and to suppress emotions instead of analysing and evaluating them adequately. This may be related to difficulties in coping with experienced emotions and an issue in realising the cause of one’s feelings (25). In turn, increased cognitive rigidity in autistic people predisposes to a literal interpretation of incoming information and adaptation problems. The pandemic situation has forced a change in lifestyle and social behaviour and staying at home, in a familiar environment. During the survey, the surveyed population must obey the sanitary regime, including: in cultural facilities such as museums, cinemas and theatres, it was possible to occupy a maximum of 50% of the seats, there was also an obligation to wear masks, in hotels it was possible to accommodate a maximum of 50% of the rooms, restaurants served only takeaway meals, water parks and gyms remained closed. The reaction of autistic people could differ from the reaction of the general population.

The aim of the present study was to determine the relationship between the severity of fear of the COVID-19 pandemic and the autistic traits in the group of participants from the general population from Poland. It also took into account the effect of individual traits of ASD, such as social skills, communication, imagination, attention to detail, attention switching/tolerance to changes, on the severity of fear.

## Methods

### Participants

The study population included adolescents and adults (the youngest participant was 14 and the oldest was 61) residing in Poland at the time of the study - 16.02.2021-11.06.2021. Of the people tested, 128 (59.81%) were women, 78 (36.44%) were men, and 8 (3.75%) people preferred not to give information about sex. Of the group of people with AQ>32, 3 (33.33%) were female, 5 (55.55%) male, and 1 (11.11%) person preferred not to give information about one’s sex. On the other hand, in those with AQ <32, 125 (60.98%) were women, 73 (35.61%) were men, and 7 (3.41%) preferred not to give information about one’s sex. The mean age in the study population was 23.78 (95%CI: 22.48-25.08). The mean age in the AQ>32 group was 18.89 (95%CI: 15.03-22.74) and in the AQ<32 group was 23.99 (95%CI: 22.66-25.33) years.

In the studied group, 56 people had primary education (26.19%), 18 people had lower secondary education (8.41%), basic vocational education – 4 people (1.87%), secondary education – 36 people (16.82%), higher education – 49 people (22.9%), and 51 people were students (23.83%). In the context of the place of residence, 27 people indicated villages (12.61%), 38 people cities up to 50 thousand inhabitants (17.75%), 28 lived in cities up to 100 thousand inhabitants (13.08%), in cities up to 250 thousand inhabitants - 49 people (22.89%), and in cities >250 thousand inhabitants - 72 people (33.64%). The demographic analysis is presented in **Table 1**.

**Table 1:** Demographic analysis of the study population.

	Study group	AQ >32
**Female**	128 (59.81%)	3 (33.33%)
**Male**	78 (36.44%)	5 (55.55%)
**Preferred not to give information about sex**	8 (3.75%)	1 (11.11%)
**Mean age**	23.78	18.89
Education		
**Primary education**	56 (26.19%)	5 (55.55%)
**Lower secondary education**	18 (8.41%)	1 (11.11%)
**Basic vocational education**	4 (1.87%)	0
**Secondary education**	36 (16.82%)	0
**Higher education**	49 (22.9%)	0
**Students**	51 (23.83%)	1 (11.11%)
Population density		
**Living in villages**	27 (12.61%)	0
**Living in cities up to 50 thousand inhabitants**	38 (17.75%)	0
**Living in cities up to 100 thousand inhabitants**	28 (13.08%)	1 (11.11%)
**Living in cities up to 250 thousand inhabitants**	49 (22.89%)	1 (11.11%)
**Living in cities >250 thousand inhabitants**	72 (33.64%)	5 (55.55%)
**Infection with SARS-CoV-2**	30 (14.02%)	2 (22.22%)

Among all of the participants, 30 (14.02%) people were infected with SARS-CoV-2 at the moment of the questionnaire or before, and 184 (85.98%) were never affected by the disease. When it comes to people with AQ ≥32, 2 (22.22%) people had COVID-19.

### Materials

The presented paper uses the Polish version of The Autism Spectrum Quotient (AQ) questionnaire, which quantifies the traits of autism (26) and the Polish version of The Fear of COVID-19 Scale (FCV-19S) questionnaire, which quantifies the severity of COVID-19 fear (27).

The Autism Spectrum Quotient has been translated and validated in the Polish population (28). It contains 5 theoretically defined subscales examining individual autistic behaviours: communication, imagination, social skills, attention switching and attention to detail. It consists of 50 questions, 10 questions for each subscale. The original cut-off point is 32 points (29).

The Fear of COVID-19 Scale has also been translated and validated in the Polish population (30). This is a short questionnaire developed at the beginning of the pandemic. It assesses emotional responses to COVID-19. It contains 7 questions, each of which is scored from 1 to 5. A score of ≥27 is defined as a high level of fear, a score of ≥20 means a moderate level of fear, a score of ≥9 means a low level of fear, a score of <9 means no association between fear and COVID-19.

### Procedures

The study was conducted between 16.02.2021 and 11.06.2021. The presented study was conducted in the form of an online questionnaire and was available via the Internet, for example, on social media websites (primarily facebook.com and instagram.com).

The inclusion criteria was age more than 13, because the version of the AQ used in this study is validated only for people of age 14 and older. Moreover, the questionnaire must have been properly filled. The exclusion criteria was only an improperly filled questionnaire.

It included 214 people with an average age of 23.78 years (95%CI: 22.48 – 25.08; max: 61, min: 14). The mean AQ score in the group was 18.65 (95%CI: 17.71 – 19.6; max: 41 min: 5). The group of people with an AQ score <32 was 205 people (95.8%), while 9 people achieved an AQ score of ≥32, which corresponds to about 4.2% of the study population.

**Figure 1:**
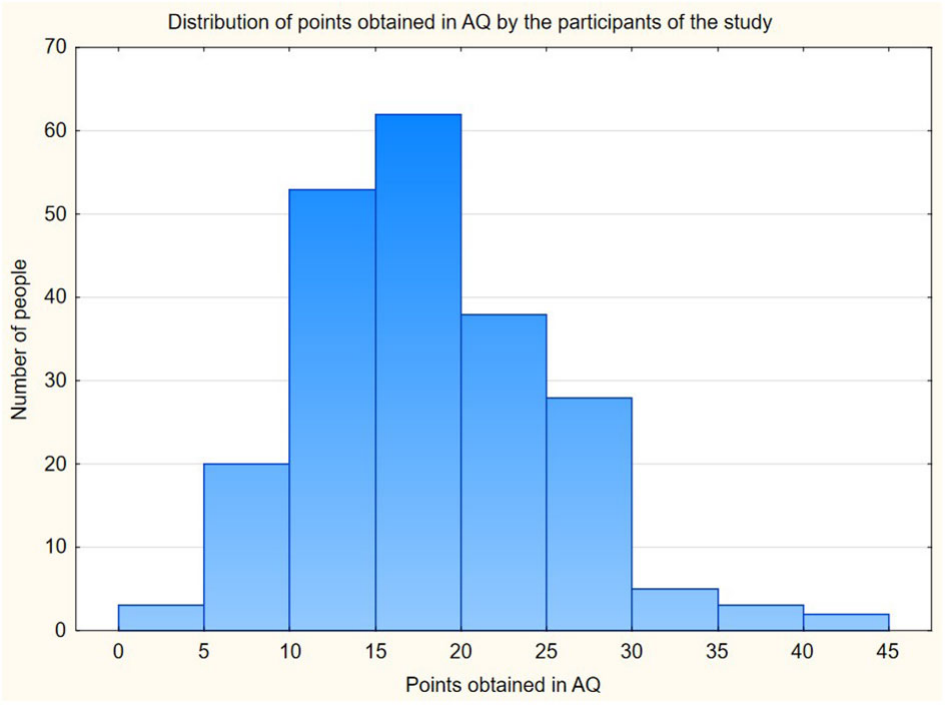
The distribution of points obtained in AQ by the participants of the study. Legend: The axis x presents points obtained in the Autism Questionnaire by the participants of the study. The axis y presents the number of people who obtained these points.

In addition, an analysis of the distribution of AQ, FCV-19S and age was performed. The Kolmogorov-Smirnov and Lillefors tests showed no statistically significant differences between the normal distribution and those observed with p=0.05 for the AQ and FCV-19S results. These types of differences were observed for the age of respondents. The distribution of AQ in the study population is present in **Figure 1**.

In the statistical calculations, StatSoft Statistica version 13 software was used. The assumed level of statistical significance was 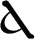
=0.05. The Spearman correlation was used to establish the relationship between AQ values x FCV-19S score, AQ subclasses x the severity of fear, age of participants x FCV-19S, education x FCV-19S, place of living x FCV-19S, a positive history of COVID-19 x FCV-19S and age of participants x AQ values. The Mann-Whitney U test was used to test the relationship between sex and FCV-19S. Multiple regression was performed using the backward stepping method. The dependent variable in both regression models was FCV-19S score and the predictors in the first regression model were AQ, age, place of living, education, SARS-Cov-2 infection in the past and sex. In the second one the dependent variables were AQ attention switching, age, AQ communication, AQ imagination, AQ social skills, AQ attention to details, sex, place of living, education and SARS CoV-2 infection in the past.

## Results

For the total sample, the fear of COVID-19 was in a moderate range (x=13.41, SD=4.9, min: 7, max: 30). A weak relationship between autistic traits and the level of fear of COVID-19 was presented in the Spearman correlation (r=0.16; p=0.01).

In addition, the analysis of the relationship between individual autistic traits and the severity of fear using the Spearman correlation showed a weak relationship between difficulties in switching attention and the severity of fear of COVID-19 (r=0.16, p=0.02). The results of the analysis of the relationship between the rest of individual autistic traits and the severity of fear using the Spearman correlation showed no relationship: between difficulties in communication and the level of fear of COVID-19 (r=0.03, p>0.05), between difficulties with imagination and the severity of fear of COVID-19 (r=-0.07, p>0.05), between problems with social skills and the intensity of fear of COVID-19 (r=0.06, p>0.05) and between troubles with attention to details and the severity of fear of COVID-19 (r=0.05, p>0.05).

In addition, the severity of fear of COVID-19 was analysed depending on other factors. First, the relationship between sex and the severity of fear of COVID-19 was analysed, showing statistically significant differences in the Mann-Whitney U test (p=0.00). The analysis excluded people who preferred not to give information about sex. The mean level of fear of COVID-19 for women was 14.2 (95%CI: 13.35 – 15.05; SD: 4.87) and for men 11.98 (95%CI: 10.93 – 13.04; SD: 4.67). Next, a correlation analysis between the level of fear of COVID-19 and age was performed using the Spearman rho test, obtaining a weak relationship between the analysed parameters (rho=0.15; p<0.05). Subsequently, the relationships with education (rho=0.03, p>0.05), place of living (rho=-0.06, p=0.42) and a positive history of COVID-19 (rho=-0.07, p>0.05) were also analysed, obtaining statistically nonsignificant differences and dependencies between the examined parameters. Moreover, we analysed the relationship between age and autistic traits using the Spearman rho test and found no statistically significant correlation (rho=-0.06, p=0.35).

Multiple regression analysis was also performed using the backward stepping method with the assumed significance level of 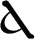
=0.05.

A normality analysis of the residuals was performed using the Kolmogorov-Smirnov test with the Lillefors correction, obtaining p=0.09, confirming the assumption that they are subject to normal distribution. The model included the AQ score and the age of the respondents and was statistically significant. The parameters of the model were equal to R^2^ = 0.1 (F=11.5; p<0.0001). The complete model is presented in **Table 2**. In the analysis of all variables – without the stepwise method – the statistical significance at α=0.1 was also used by the parameter “sex” with a low, negative parameter ß=-0.12.

**Table 2:** Multiple regression regarding factors influencing the severity of fear according to FCV-19S test results.

Independent variables	b	std. err of b	t (207)	p
Constant	10.88	2.69	4.04	0.00008
**AQ**	**0.12**	**0.05**	**2.53**	**0.01**
**Age**	**0.15**	**0.04**	**3.91**	**<0.001**
Place of living	-0.19	0.23	-0.86	0.39
Education	-0.19	0.19	-0.99	0.32
SARS-CoV-2 infection in past	-0.21	0.94	-0.22	0.83
Sex	-1.06	0.58	-1.83	0.07

The bold values mean that they are statistically significant.

A second multiple regression analysis was also performed using the backward stepping method with the assumed significance level of 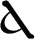
=0.05. The model included the subclasses of AQ score and the age of the respondents. The model was statistically significant. The parameters of the model were equal to R^2^ = 0.1 (F=14.73; p<0.000001). The model showed the influence of AQ attention switching (p=0.0004) and age (p=0.00001) on the level of felt fear of COVID-19. The complete model of this regression analysis is presented in **Table 3**.

**Table 3:** Multiple regression regarding factors influencing the severity of fear according to FCV-19S test results (with subclasses of AQ).

Independent variables	b	std. err of b	t (207)	p
Constant	10.8	2.75	3.92	0.0001
**AQ attention switching**	**0.46**	**0.17**	**2.75**	**0.006**
**Age**	**0.17**	**0.03**	**4.43**	**0.00002**
AQ communication	0.3	0.22	1.44	0.15
AQ imagination	-0.11	0.2	-0.54	0.59
AQ social skills	-0.05	0.17	-0.28	0.78
AQ attention to details	-0.11	0.15	-0.74	0.46
Sex	-0.86	0.58	-1.48	0.14
Place of living	-0.2	0.22	-0.88	0.38
Education	-0.26	0.19	-1.37	0.17
SARS-CoV-2 infection in past	-0.29	0.94	-0.31	0.76

The bold values mean that they are statistically significant.

## Discussion

The aim of the present study was to determine the relationship between the severity of fear of the COVID-19 pandemic and the autistic traits in the group of participants from the general population from Poland. It also took into account the effect of the individual traits of ASD, such as social skills, communication, imagination, attention to detail, attention switching/tolerance to changes, on the severity of fear.

Since autistic traits are continuum, even for the people from the general population without the diagnosis of ASD, the higher the level of autistic traits they have, the higher the likelihood of difficulties characteristic for ASD is. ASD characteristic’s feature is increased cognitive stiffness and difficulties in relating the information obtained to the context. Media reports on COVID-19, charged with a large dose of sensation, in the perspective of this type of cognitive deficits, are material difficult to clear from the emotional load. For this reason, people with ASD may have difficulty processing information on this subject, which in turn may lead to incorrect reconciliation of the level of fear with the actual level of threat, which will significantly influence the functioning. In fact, the study population showed an effect of the difficulties with attention switching (one of the autistic traits) on the degree of fear of the COVID-19 pandemic. Of course, the obtained regression parameters indicate a small impact, which suggests that this is one of many factors determining the response to such threats. The psychological response to stimuli that induce high levels of stress is conditioned by many different factors. Functional disorders can take the form of chronic stress, delayed reaction to stress that decreases over time or resistance to stress. An individual’s emotional response to stress is largely determined by perceived or actual exposure to stress, but also by a person’s internal resources and external resources. Factors conducive to resistance to the perceived high level of fear, anxiety and stress include optimism, social support, the ability to critically assess the media message, reducing the feeling of social isolation, especially through online communication during the pandemic. At the level of family relationships, adaptability, family cohesion, good communication and proper financial management play an important role (31). A factor considered to have a particularly protective effect during stressful situations is having an external source of social support. Positive high-quality social support can increase resistance to stress, help protect the development of trauma-related psychopathology and reduce the functional consequences of trauma-induced disorders (32). In contrast, strong predictors of trauma-induced disorders are a lack of social support, low intelligence and a lack of education, positive family history, previous psychiatric history, and aspects of the trauma response itself, such as dissociative responses (33). An important factor that increases the likelihood of developing a high level of stress is also the intensification of self-criticism and low self-esteem with insufficient ability to feel self-compassion, i.e. kindness towards oneself when experiencing disappointments and painful life experiences (34). Also, the history of traumas experienced so far and difficult life situations, especially those experienced during childhood, is not without significance. The brain is characterised by the greatest plasticity in childhood and adolescence. While it has many benefits, allowing children to learn quickly from experiences and adapt to the environment in which they are raised, it can also have long-term consequences for children raised in adverse conditions. In particular, environments characterised by violence and a high potential for harm can influence social, emotional and neurobiological development patterns in a way that enables the rapid detection of potential threats. Although these developmental adaptations may increase safety in hazardous environments by mobilising behavioural responses to avoid threats, they may also increase the risk of many forms of psychopathology (35).

According to the adopted hypothesis that the level of autistic traits have effect on the level of fear of the COVID-19 pandemic, the obtained results also indicate that difficulties with attention switching and connected with that difficulties with regulation of emotions are the areas that most strongly determine the subjective sense of fear in the face of danger in people with higher autistic traits scores. Parameters that are less important or do not affect level of fear are impaired ability to see a holistic picture of the situation, low awareness of the potential consequences, problems with social interaction, and communication. Similar results were also obtained in the studies of other authors. Hollock et al. (36) have shown that cognitive inflexibility can be an important factor associated with emotional difficulties. Thus, patients with higher levels of autistic traits who show greater cognitive stiffness than those with lower levels of autistic traits may experience co-occurrence of emotional and behavioural difficulties. The association of cognitive rigidity with level of fear may result from reduced tolerance of uncertainty, which has a documented association with anxiety and fear levels in children with ASD (37). Higher levels of fear can be associated with disturbances in the effective regulation of emotions as well. Ineffective coping mechanisms for a variety of emotional states in response to stress stimuli can lead to inappropriate behavioural responses in people with ASD. Insufficient innate ability to regulate emotions is also associated with deficits in the area of theory of mind, i.e. the ability to cognitively and affectively accept other people’s perspectives and understand their intentions, as well as recognise their own state of mind (38). Regardless of this, according to literature data, patients with ASD experience all emotions, including anxiety, more strongly (39), hence they may have a higher severity of fear of the COVID-19 pandemic than in the general population. People with ASD suffer from difficulties in adapting to the changing environment around them (40), which in the face of sudden changes caused by the start of the pandemic may have exacerbated their fear. The ability to switch attention is a factor in reducing the level of anxiety and fear (41), while less effectiveness in its range predisposes to a higher level of anxiety and fear (42). Appropriate mindfulness and concentration exercises reduce the symptoms of anxiety while increasing the flexibility of attention (43).

For this reason, it is necessary to take care of the population of people with higher autistic traits by creating programs aimed at teaching concentration, attention and coping with stressful situations. Studies have found that people with higher levels of autistic traits are more sensitive to the psychosocial effects of the pandemic, such as anxiety, depression, financial problems, job loss or marital problems. Rapidly changing social and environmental conditions, such as the beginning of the COVID-19 pandemic, point to the need to increase access to psychological and psychiatric care. It is also important to note that developing mental health problems in people with higher autistic traits can increase the time to contact with healthcare and to receive optimal help. Health system management bodies must correctly recognise the complex problems of this group of patients and the psychological losses they suffer. The overriding goal should be to minimise and alleviate their discomfort, as well as to facilitate the return to the previous level of functioning in society. In order to better deal with these types of psychosocial problems affecting people with higher levels of autistic traits, the government and health professionals should create an appropriate model for intervention and prevention of psychosocial crises. The needs of people with higher degree or autistic traits or ASD are different from those of the general population, and health systems governing bodies should provide them with the support and appropriate care that could be crucial in the next peaks of pandemics, crises and disasters around the world. The law should guarantee additional freedoms for patients with ASD in crisis situations, but at the same time they should ensure their safety (44). Guidelines and recommendations for protecting the mental health of people with ASD in the face of a pandemic are well known in the scientific community (45), but they should also be disseminated in the general population, especially in health care facilities and among bodies responsible for setting up support programmes for people with higher autistic traits. It seems a good idea to replace face-to-face communication with online contact during periods of difficult social interaction, as well as training programs for relatives of ASD patients so that they can be able to provide patients with the necessary support to cope with stressful situations (46).

In the present study, there is an overrepresentation of people with higher autistic traits according to the results they obtained in AQ (≥32), whose percentage was equal to about 4.5% of respondents. This is more than four times higher than the estimated population frequency worldwide, which is about 1% (47). This difference is probably the result of the methodology used, i.e. a questionnaire survey conducted by social media. They allow reaching different groups of people through platforms that often suggest topics to the user and groups similar to your search history and interests. The forms provided by us were found not only on social profiles, but also on groups and websites devoted to the above-mentioned disorders. Because of this, the recipients of the questionnaire were people interested in the topic, some could suspect the presence of ASD, which may have encouraged them to take part in the study. As shown in the study Jurek et al. (48), people with ASD are particularly involved in internet use, which allows them to develop interests related to a specific topic and to make friends with people who share these interests. This may also explain the increased interest in the survey.

As the obtained data shows, there is a positive correlation between age and the level of fear of the COVID-19 pandemic. These results are consistent with other studies (12). Higher levels of fear may be caused by a greater awareness of the possible consequences of the pandemic and a better understanding of the situation. With age, the level of fear about one’s own health increases (49). The older the person, the higher the level of fear of death is felt by him (50). COVID-19 is a life-threatening disease in every age group, and more mature people have a more responsible approach to the situation. People in older age groups experience more stressors in everyday life not only in terms of health, but also socioeconomic and educational, which has been highlighted by the pandemic and increased the overall level of fear among them.

This study did not show a statistically significant relationship between the population density in the place of residence, education, SARS-CoV-2 infection and the severity of fear of the COVID-19 pandemic. The limitation of the study was the homogeneity of the group in terms of the above-mentioned features. Another limitation of the study is an overrepresentation of people with AQ ≥32 as it’s a disturbance in relation to the general population. We decided to distribute the questionnaire in social media and, as stated before, people who are more interested in the ASD topic, are more eager to fill such a questionnaire, as they might suspect ASD in themselves. Although it’s also important to highlight that the survey group does not have a clinical diagnosis of ASD. Next limitation is that the data was collected through an online survey and reached a limited number of respondents. Additionally, the data were collected during the third wave of the pandemic and people knew more about the virus then. They might have felt less fear, as the vaccine had already been distributed.

Overall, the results of the present study showed the impact of the autistic traits on the level of fear of the COVID-19 pandemic. There is a relationship between the symptoms associated with attention switching and the fear felt. It also increases with age. This suggests that people who are more prone to a higher level of fear require more attention during new, stressful situations. These findings are consistent with previous studies that people experiencing higher levels of anxiety and fear are those with special needs during the pandemic and stressful situations (11). Appropriate patterns of attention and interpretation of events may be helpful in dealing with situations that arouse great emotions (50). Establishing a routine for people with ASD may lead to reducing the experienced level of fear (12).

## Conclusions

1. The autistic traits affect the level of fear of the COVID-19 pandemic.

2. Of particular importance to the level of fear of the COVID-19 pandemic is the intensity of symptoms in the field of the ability to switch attention.

3. In the study population, there is a positive correlation between age and the level of fear of the COVID-19 pandemic.

4. The population of people with higher autistic traits or ASD requires special attention from health system management bodies in crisis situations.

## Data availability statement

The raw data supporting the conclusions of this article will be made available by the authors, without undue reservation.

## Ethics statement

The research was conducted in accordance with related laws, regulations, and guidelines on research ethics (USTAWA z dnia 5 grudnia 1996 r. o zawodach lekarza i lekarza dentysty). The consent to participate in the survey was obtained.

## Author contributions

DB: Investigation, Writing – original draft, Writing – review & editing. AŚ: Investigation, Writing – original draft, Writing – review & editing. AB: Investigation, Writing – original draft, Writing – review & editing. FT: Investigation, Writing – original draft, Writing – review & editing. KW: Conceptualization, Investigation, Methodology, Supervision, Writing – review & editing. MJ-K: Conceptualization, Investigation, Methodology, Supervision, Writing – review & editing.
